# Energy-Efficient Configuration and Control Allocation for a Dynamically Reconfigurable Underwater Robot

**DOI:** 10.3390/s23125439

**Published:** 2023-06-08

**Authors:** Tho Dang, Lionel Lapierre, Rene Zapata, Benoit Ropars

**Affiliations:** 1 Laboratory of Informatics, Robotics and MicroElectronics (LIRMM) (UMR 5506 CNRS—UM), Université Montpellier, 161 rue Ada, CEDEX 5, 34392 Montpellier, France; zapata@lirmm.fr; 2 Reeds Company, 199 rue Hélène Boucher, 34170 Castelnau-Le-Lez, France; ropars@lirmm.fr

**Keywords:** autonomous underwater robot, dynamically reconfigurable underwater robot, control allocation, optimization

## Abstract

A dynamically reconfigurable underwater robot, which can vary its configuration during a mission, would be useful for confined environment exploration and docking because of its versatility. A mission can be performed by choosing among different configurations, and the energy cost may increase, owing to the reconfigurability of the robot. Energy saving is the critical issue in long-range missions with underwater robots. Moreover, control allocation must be considered for a redundant system and input constraints. We propose an approach for an energy-efficient configuration and control allocation for a dynamically reconfigurable underwater robot that is built for karst exploration. The proposed method is based on sequential quadratic programming, which minimizes an energy-like criterion with respect to robotic constraints, i.e., mechanical limitations, actuator saturations, and a dead zone. The optimization problem is solved in each sampling instant. Two popular tasks for underwater robots, i.e., path-following and station-keeping (observation) problems, are simulated, and the simulation results show the efficiency of the method. Moreover, an experiment is carried out to highlight the results.

## 1. Introduction

### 1.1. A Dynamically Reconfigurable Underwater Robot and Perspectives

In robotic fields, reconfigurable robots are an attractive area because of their versatility. They can change their shape or configuration corresponding to specific mission requirements. Therefore, the building cost can be reduced by one robot performing several tasks. Moreover, reconfigurable robots can be applied for complex tasks requiring adaptive configurations, such as karst exploration or space applications. Robustness is also an advantage of reconfigurable robots in terms of the flexibility. Overviews of these aspects and other issues of modular self-reconfigurable robot systems are available in the literature [1,2].

**Figure 1:**
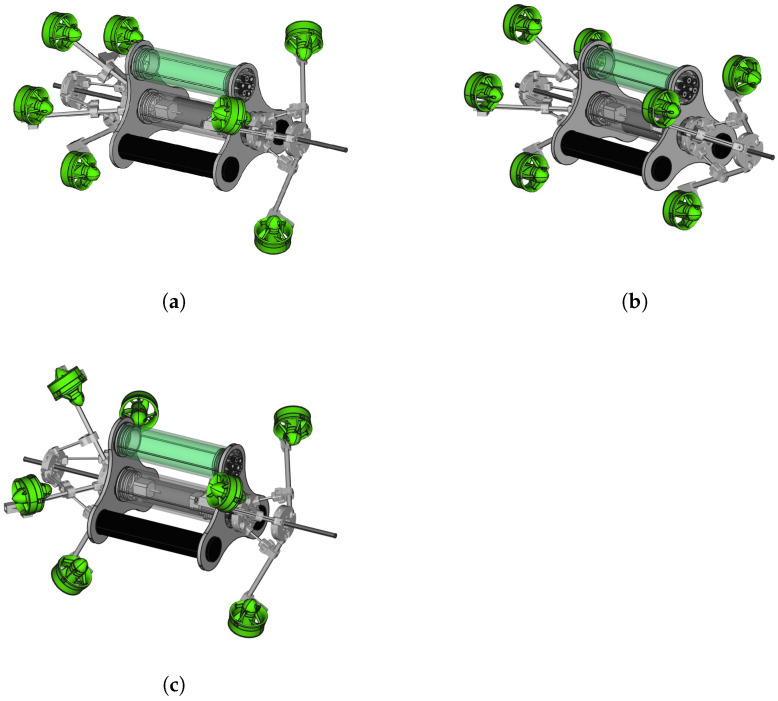
A 3D model of dynamically reconfigurable robot with three different configurations [3]. (**a**) Robot in open–close state. (**b**) Robot in close–close state. (**c**) The robot in open–open state.

A dynamically reconfigurable underwater robot was built in our laboratory at Montpellier University. Readers can refer to [3] for more details. The robot consists of seven thrusters (three forward and four backward thrusters), and its configuration can be dynamically varied. Figure 1 shows some configurations of the robot, i.e., the robot has an open state for the forward branch and a close state for the backward branch, called an open–close state. The similarities are shown in Figure 1b,c, corresponding to close–close and open–open states, respectively. The change in the robot configuration can be viewed at https://youtu.be/yBBCu1z3q-0 (accessed on 7 February 2023). In the close–close state, it operates as a torpedo-shaped robot; in the open–open state, it operates as an isotropic system. Our robot can operate as an over-actuated system.

One of the challenges of an over-actuated system is control allocation, in which the distribution of the applied forces on actuators, the so-called applied force vector, is found when a desired control vector (output from a controller) is given. In the following, we describe control allocation methods for an over-actuated system.

### 1.2. Control Allocation

The basic property of an over-actuated system is that the number of actuators is larger than the controllable degrees of freedom (DOFs). The problem is how to map the desired actuation on the DOFs to forces on the actuators through a *configuration matrix*. In the literature, two approaches are developed to solve this problem. The first method is to divide the control design into two levels. In the first level, the control laws for each DOF are designed. The outputs of this level, called the desired control vector, are the inputs of the second level. In the second level, a control allocation algorithm is designed to assign the control inputs for actuators to optimize one or some cost functions with respect to redundancy and actuator limitations. The problem at the second level is called the control allocation (CA) problem. With the second method, the control inputs (normally with constraints) are directly considered in the control design process. This issue arises in the model predictive control (MPC) method because control allocation is considered a constraint in the MPC formulation. However, this increases the computational cost, so it is the most challenging issue in the MPC problem.

**Figure 2:**
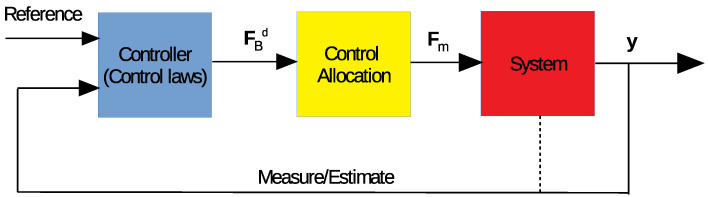
Control allocation block in general control loop.

The control allocation problem is one of the main tasks in the control design of over-actuated or redundant systems. Normally, the actuators of a system are constrained with mechanical and electrical limitations, such as saturation or a dead zone. The role of the control allocation block in a control loop is displayed in Figure 2, in which the input is the desired control vector (F${}_{B}^{d}$), and the output is the applied force vector (F${}_{m}$).

The many available control allocation methods are divided into two groups: pseudo-inverse- and optimization-based methods, and with or without constraints. Without constraints, the problem is easier. However, the unconstrained control allocation problem provides the basic ideas for many constrained control allocation problems. Most of the control allocation methods are based on optimization techniques, either explicitly or implicitly. Depending on the application, the appropriate control allocation method is chosen.

Some surveys have been conducted on the control allocation problem in recent years. In [4], the authors compared many control allocation algorithms with closed- and open-loop measures. In [5], the authors evaluated the performance and computational cost of the optimization methods of the control allocation problem. In [6,7], control allocation methods for ships and underwater vehicles were investigated. A survey was published in 2013 [8], in which many control allocation methods and applications were presented and discussed. With the advances in neural networks (NNs), NN-based control allocation approaches have been developed [9,10].

### 1.3. The Singularity of Control Allocation

As mentioned above, many approaches can be used to solve the CA problem. However, in most cases, a configuration matrix is constant and remains unchanged during the robot operation. When the configuration matrix is varied, it may yield a singular or a near-singular configuration; therefore, some DOFs are not controllable. This was previously discussed [11] by researchers who proposed an approach to penalize the singularity of the configuration. However, some advantages of the singular configuration have been addressed, such as when facing disturbances and to achieve optimal energy. Owing to [12], we can investigate different control allocation methods for near-singular configurations. In this situation, the minimum singular value of the configuration matrix is too small. This yields a pseudo-inverse that is too large (which is easily observed with the singular value decomposition of the configuration matrix) and causes a large error if pseudo-inverse-based CA methods are used. Hence, optimization-based CA methods are suitable for dynamically reconfigurable robots.

### 1.4. Control Allocation with Varying Configuration Matrix

Control allocation methods with a varying configuration matrix have been introduced [13,14,15] for fault tolerance, in which the control performance is guaranteed when the efficiency of the actuators is lost. In another direction, the configuration matrix contains variables that must be found to minimize an objective function, which is normally power consumption. In the literature, this concept was only implicitly introduced in one study [11], in which azimuths were found to optimize energy consumption. In this study, the problem was formulated and approximated as locally convex quadratic programming. In particular, a sack variable was added to guarantee that the optimization problem always had a feasible solution and, in each sample, the nontrivial part (updating part) of the optimization problem was found by linearizing the objective function and constraints on the optimal solution of the previous sample.

For dynamically reconfigurable robots, the configuration matrix can be varied during robot operations. One question is how to achieve an optimal energy criterion with respect to the parameters of the configuration matrix and the control allocation problem. Motivated by such robots, a real-time technique in nonlinear MPC, so-called one-iteration optimization [16], and optimization-based control allocation methods, we developed an approach to achieve energy-efficient configuration and control allocation, which is different from the aforementioned method, for a dynamically reconfigurable robot that can vary its configuration during missions. The main contributions of the paper are summarized as follows:

1. Propose an energy-efficient configuration problem for a dynamically reconfigurable robot with respect to its constraints.
2. Propose an integration of a one-iteration optimization technique and a control allocation method to solve the energy-efficient configuration problem.

The remainder of this paper is organized as follows: basic notations are summarized in Appendix B. The energy-efficient configuration and control allocation problem are presented in Section 2. A proposed solution is introduced in Section 3. The simulation results are shown and discussed in Section 4. The experimental result is discussed in Section 5; finally, concluding remarks are provided in Section 6.

## 2. Energy-Efficient Configuration and Control Allocation Problem

**Figure 3:**
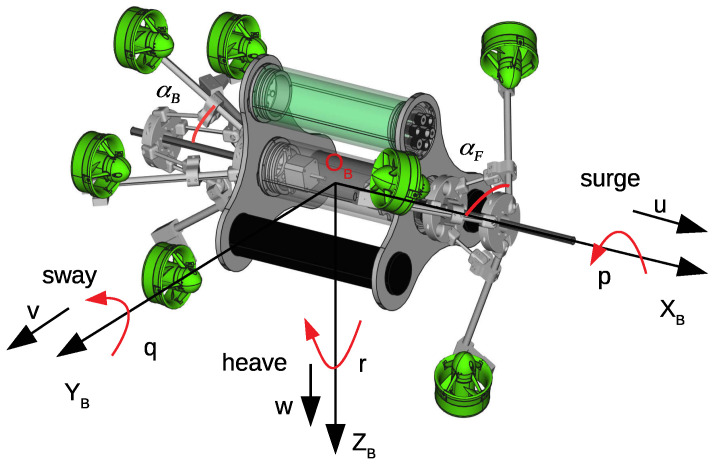
Notations and definitions of two angles $\alpha_{F}$ and $\alpha_{B}$.

Without loss of generality and to ensure ease of understanding, our robot is used to formulate the problem. Some additional notations are illustrated in Figure 3, i.e., body frame, $X_{B},Y_{B},Z_{B}$; linear velocities and angular rates expressed in body frames u,v,w and p,q,r, respectively; and two angles $\alpha_{F},\alpha_{B}$, for changing the robot configuration, which can be changed during robot operations.

The relationship between the resulting control vector, including force and torque elements, in the body frame, denoted as F${}_{B}$, and the force vector applied on the thrusters, denoted as F${}_{m}$, is described as a mapping through the *configuration matrix*, denoted as A, which describes the geometric organization of thrusters in the body frame:

(1) FB=A(αF,αB)Fm=fτ

where $F_{B}\in R^{6}$, $A\in R^{6\times m}$, $F_{m}={\left[F_{m,1}\;F_{m,2}\;...\;F_{m,m}\right]}^{T}\in R^{m}$, and *m* is the number of thrusters, m=7>6. Because the system has 6 DOFs with 7 actuators, the actuation system is said to be redundant or over-actuated.

From the scheme of the robot, the configuration matrix is as follows:

(2) $$\begin{array}{c} \begin{array}{cc} A\; & =\left(\begin{array}{cccc} u_{1}^{B} & u_{2}^{B} & \cdots & u_{m}^{B}\\ r_{1}^{B}⊗u_{1}^{B} & r_{2}^{B}⊗u_{2}^{B} & \cdots & r_{m}^{B}⊗u_{m}^{B} \end{array}\right)\\ \; & =\left(\begin{array}{cccc} u_{1}^{B} & u_{2}^{B} & \cdots & u_{m}^{B}\\ \tau_{1}^{B} & \tau_{2}^{B} & \cdots & \tau_{m}^{B} \end{array}\right)=\left(\begin{array}{c} A_{1}\\ A_{2} \end{array}\right) \end{array} \end{array}$$

where m=7 and $u_{1}^{B},\ldots ,u_{7}^{B}$ and $r_{1}^{B},\ldots ,r_{7}^{B}$ are shown in Appendix A. The basic idea for the computing matrix A is to use transformation matrices between the coordinate systems. Because of space limitations, this computation is not shown in this paper. When two angles, $\alpha_{F}$ and $\alpha_{B}$ (Figure 3), are varied, the robot’s configuration (A matrix) changes.

In this section, we consider an energy-efficient configuration and the control allocation problem, in which the objective function is defined using the Euclidean norm of the applied force vector, $F_{m}$ with respect to mechanical constraints: the thruster limitations. In particular, the problem is formulated as

(3a) $$\begin{array}{cc} \min_{\alpha_{F},\alpha_{B},F_{m}} & J=∥F_{m}{∥}^{2} \end{array}$$

(3b) $$\begin{array}{cc} s.t & \;45^{\circ }\leq \alpha_{F},\alpha_{B}\leq 90^{\circ } \end{array}$$

(3c) $$\begin{array}{cc} \; & \;F_{m}\in F \end{array}$$

(3d) $$\begin{array}{cc} \; & \;F_{B}^{d}-A\left(\alpha_{F},\alpha_{B}\right)F_{m}=0 \end{array}$$

where $F_{B}^{d}$ is the desired control vector (output from the controller), and F is a feasible set of thruster forces. The constraint (3b) is the mechanical limitation on the robot, in which two angles can vary from $45^{\circ }$ to $90^{\circ }$.

The objective function is chosen as the Euclidean norm of the applied force vector. This is reasonable because of the nearly linear characteristics of the thrusters used in our robot (see more details in the simulation section). The problem objective is to find two angles, $\alpha_{F},\alpha_{B}$ and applied force vector $F_{m}$, to minimize function *J* and to satisfy the constraints. This is a nonlinear optimization problem that is solved at each sampling time (called online optimization) because the desired control vector $F_{B}^{d}$ is changed in each time step in the general case. Note that in our problem, the configuration matrix A is dynamic and has two angles, $\alpha_{F}$ and $\alpha_{B}$ (see Figure 3).

Other perspectives we needed to consider are the reactivity of the robot (the time for state propagation or system response) and the time delay of the changing configurations. If the system response is too fast, we cannot apply online optimization. Our objective was to solve the online optimization problem; therefore, we assumed that the time required for solving at least one iteration of the optimization problem is less than the time that the system needs from the current to the next state. In our case of an underwater robot, this assumption is reasonable.

For the time delay when changing configurations, assume that at time step *k*, we have two angles: $\alpha_{Fk}$ and $\alpha_{Bk}$. At the next time step, k+1, assume that we obtain a solution from the optimization problem with two angles $\alpha_{F(k+1)}$ and $\alpha_{B(k+1)}$. Physically, the robot requires time, $△t_{\alpha }$, to change from $\alpha_{Fk}$ to $\alpha_{F(k+1)}$ and from $\alpha_{Bk}$ to $\alpha_{B(k+1)}$, which is the new optimal configuration. This changing time cannot be too fast, given the limitations of the DC motors used for changing the robot configuration. However, this change must be completed before the next time step, k+1. If not, the configuration matrix will not be associated with the correct corresponding time step k+1. In other words, the time needed for changing, $△t_{\alpha }$, must be less than the sampling time. Therefore, the consecutive values of these two angles must be small enough.

To solve our problem, two assumptions were applied, as follows:

**Assumption** **A1.** *The reactivity of the system is long enough, i.e., to solve the online optimization problem and basic mathematical operations. In particular, the sampling time of our underwater robot is 0.1 (s). The computational time of one-iteration optimization and other basic mathematical operations is in centiseconds, e.g., 0.01 (s) (this depends on the computation system) and the system reactivity of an underwater robot, which normally depends on its shape and drag coefficients, is also in centiseconds.*

**Assumption** **A2.** *The time for changing the mechanical system between two consecutive angles is fast enough in one sampling time.*

In practice, Assumption 1 is reasonable for underwater robots. Assumption 2 can be satisfied if the derivation between two consecutive angles is small enough.

**Remark** **1.** *The problem (3) is a nonconvex parametric optimization problem, which has to be solved at each sampling time. Thus, as obtaining exact optimal solution is too challenging or probably impossible, the idea is to find an approximate solution.*

**Remark** **2.** *The deviation of two angles $\alpha_{F},\alpha_{B}$ in two consecutive time steps is small enough, owing to mechanical and electrical limitations and Assumption 2.*

## 3. Solution

The procedure of finding the approximate solution of the problem is divided into two steps: *Predictor* and *corrector*. In the *predictor* step, the problem is quickly solved (in one iteration) to obtain the approximately optimal two angles and applied force vector. However, the hard constraint (3d) cannot be easily satisfied with this applied force vector. With these two angles, we can instantly compute the configuration matrix and move to the next step, *corrector*. In this step, an algorithm is used to find a better applied force vector with respect to this hard constraint. The two following subsections describe some basic results from real-time model predictive control, which we used in the *predictor* step.

### 3.1. Sequential Quadratic Programming (SQP)

Consider a nonlinear optimization problem (NLP):

(4a) $$\begin{array}{cc} \min_{x} & \;J(x) \end{array}$$

(4b) $$\begin{array}{cc} s.t\; & G(x)=0 \end{array}$$

(4c) $$\begin{array}{cc} \; & H(x)\geq 0 \end{array}$$

Sequential quadratic programming (SQP) is an iterative method to find a Karush–Kuhn–Tucker (KKT) point of an NLP. In particular, starting with an initial guess $y_{0}=\left(x_{0},\lambda_{0},\mu_{0}\right)$ (where $x_{0}$ is a primal variable, and $\lambda_{0},\mu_{0}$ are Lagrangian multipliers), an SQP method iterates:

(5) $$y_{k+1}=y_{k}+\delta_{k}\Delta y_{k}$$

where $\delta_{k}\in \left(0,1\right]$, and

(6) $$\Delta y_{k+1}=\left(\begin{array}{c} \Delta x_{k}\\ \Delta \lambda_{k}\\ \Delta \mu_{k} \end{array}\right):=\left(\begin{array}{c} \Delta x_{k}\\ \tilde{\lambda_{k}}-\lambda_{k}\\ \tilde{\mu_{k}}-\mu_{k} \end{array}\right)$$

Equation (6) is obtained from the solution point $\left(\Delta x_{k},\tilde{\lambda_{k}},\tilde{\mu_{k}}\right)$ of the following quadratic programming:

(7a) $$\begin{array}{cc} \min_{\Delta x\in \Omega_{k}} & \;\frac{1}{2}\Delta x^{T}A_{k}\Delta x+\nabla_{x}J{\left(x_{k}\right)}^{T}\Delta x \end{array}$$

(7b) $$\begin{array}{cc} s.t\; & G\left(x_{k}\right)+\nabla_{x}G{\left(x_{k}\right)}^{T}\Delta x=0 \end{array}$$

(7c) $$\begin{array}{cc} \; & H\left(x_{k}\right)+\nabla_{x}H{\left(x_{k}\right)}^{T}\Delta x\geq 0 \end{array}$$

Readers can refer to [17] for more details. The choice of the step length $\delta_{k}$, Hessian matrix $A_{k}$, and set $\Omega_{k}\subset R^{n_{x}}$ derives variants of existing SQP methods. If we choose $\delta_{k}:=1,\Omega_{k}:=R^{n_{x}}$ and the Hessian matrix as in Equation (8), this is a full-step exact Hessian SQP method, which is appealing and important [18]:

(8) $$A_{k}:=\nabla_{x}^{2}L\left(x_{k},\lambda_{k},\mu_{k}\right)$$

This choice has an advantage, in that the full-step exact Hessian SQP method shows the same high-quality local convergence behavior as the Newton–Raphson method in the vicinity of a solution of the KKT system (for an illustration for cases with equality constraints, see [17]). Note that a good initial guess is required not only for the primal variable x but also for the multipliers λ,μ (for a proof, refer to [19]).

### 3.2. Parametric Nonlinear Optimization

In this section, we describe a parametric optimization problem, P(t), in which a parameter is changed as follows:

(9a) $$\begin{array}{cc} \min_{x} & \;J(x,t) \end{array}$$

(9b) $$\begin{array}{cc} s.t\; & G(x,t)=0 \end{array}$$

(9c) $$\begin{array}{cc} \; & H(x,t)\geq 0 \end{array}$$

where t is a parametric variable. Note that this is not a time variable.

The problem (9) is equivalent to the following problem $P(\bar{t})$:

(10a) $$\begin{array}{cc} \min_{x,t} & \;J(x,t) \end{array}$$

(10b) $$\begin{array}{cc} s.t\; & t-\bar{t}=0 \end{array}$$

(10c) $$\begin{array}{cc} \; & G(x,t)=0 \end{array}$$

(10d) $$\begin{array}{cc} \; & H(x,t)\geq 0 \end{array}$$

The problem (10) is only different from (9) by reforming variable t, which is fixed as an additional constraint $t-\bar{t}=0$. This is introduced to show that *the first iteration of the SQP approach of the problem is the first-order approximation of the solution manifold of this problem*. The following theorem is extracted to highlight this point. Readers can refer to [18] for the proof and more details.

**Theorem** **1** (First-order prediction by exact Hessian SQP [18]). Let us assume that we found a KKT point $\left(x^{*}\left(0\right),\lambda^{*}0,\mu^{*}\left(0\right)\right)$ of problem P(0) that satisfies the sufficient optimality conditions. If a full-step SQP algorithm with an exact Hessian for the solution of the problem P(ϵ), with ϵ>0 being sufficiently small, is started with this solution as an initial guess, then the nontrivial part of the first SQP step (Δx,Δλ,Δμ) is identical to ϵ times the one-sided derivative of the solution manifold $\left(x^{*}\left(t\right),\lambda^{*}\left(t\right),\mu^{*}\left(t\right)\right)$ of the problem P(t), i.e.,

(11) $$\begin{array}{c} \lim_{t\to ,t>0}\frac{1}{t}\left(\begin{array}{c} x^{*}\left(t\right)-x^{*}\left(0\right)\\ \lambda^{*}\left(t\right)-\lambda^{*}\left(0\right)\\ \mu^{*}\left(t\right)-\mu^{*}\left(0\right) \end{array}\right)=\left(\begin{array}{c} \delta x\\ \delta \lambda \\ \delta \mu \end{array}\right)=\frac{1}{\epsilon }\left(\begin{array}{c} \Delta x\\ \Delta \lambda \\ \Delta \mu \end{array}\right) \end{array}$$

### 3.3. Online Optimization Observation in SQP

Following Theorem 1, an observation is that the first quadratic programming solution of a full-step exact Hessian SQP algorithm provides a good approximation of the exact solution if the algorithm is initialized in a neighborhood of this solution. In an online optimization scenario, it would probably be better to use an approximation of the first correction instead of waiting until the SQP algorithm converges. After the first SQP iteration, we correct the solution if disturbances exist that affect the initial guess or violate the constraints; if no further disturbance occurs, the algorithm can continue to improve the outcome of the previous iterations. In our case of an energy-efficient configuration of the robot, the change in the two angles $\alpha_{F},\alpha_{B}$ in each time step is small. Hence, from an initial guess, two angles can be chosen after one iteration of SQP. After that, we can correct the applied force vector to obtain a better solution.

### 3.4. Algorithm

#### 3.4.1. *Predictor* Step

This problem (3) can be rewritten as

(12a) $$\begin{array}{cc} \min_{\alpha_{F},\alpha_{B},F_{m},F_{B}} & J=∥F_{m}{∥}^{2} \end{array}$$

(12b) $$\begin{array}{cc} s.t & \;45^{\circ }\leq \alpha_{F},\alpha_{B}\leq 90^{\circ } \end{array}$$

(12c) $$\begin{array}{cc} \; & \;F_{m}\in F \end{array}$$

(12d) $$\begin{array}{cc} \; & \;F_{B}-A\left(\alpha_{F},\alpha_{B}\right)F_{m}=0 \end{array}$$

(12e) $$\begin{array}{cc} \; & \;F_{B}-F_{B}^{d}=0 \end{array}$$

where $F_{B}$ is a parametric variable, and $F_{B}^{d}$ is its constraint and is changed during a time instant (output from a controller). This problem has the form of problem (10) and is solved in one iteration using SQP.

By denoting $x={\left[\alpha_{F}\;\alpha_{B}\;F_{m}\right]}^{T}$, the problem (12) can be formed as

(13a) $$\begin{array}{cc} \min_{x,F_{B}} & \;J(x) \end{array}$$

(13b) $$\begin{array}{cc} s.t & \;G(x,F_{B})=0 \end{array}$$

(13c) $$\begin{array}{cc} \; & \;H(x)\leq 0 \end{array}$$

(13d) $$\begin{array}{cc} \; & \;F_{B}-F_{B}^{d}=0 \end{array}$$

where $F_{B}$ replaces the role of t and $F_{B}^{d}$ replaces the role of $\bar{t}$ in problem $P(\bar{t})$.

The quadratic programming used to find direction in SQP, $\Delta x_{k}$, $\Delta F_{B}$, is as follows:

(14a) $$\begin{array}{cc} \min_{\Delta x,\Delta F_{B}} & \frac{1}{2}\Delta x^{T}\nabla_{x}^{2}L\Delta x+\nabla_{x}J^{T}\Delta x+\nabla_{F_{B}}J^{T}\Delta F_{B}+\frac{1}{2}\Delta F_{B}^{T}\nabla_{F_{B}}^{2}L\Delta F_{B}+\Delta F_{B}^{T}\nabla_{x,F_{B}}^{2}L\Delta x \end{array}$$

(14b) $$\begin{array}{cc} s.t & \;G+\nabla_{F_{B}}G^{T}\Delta F_{B}+\nabla_{x}G^{T}\Delta x=0 \end{array}$$

(14c) $$\begin{array}{cc} \; & \;H+\nabla_{F_{B}}H^{T}\Delta F_{B}+\nabla_{x}H^{T}\Delta x\leq 0 \end{array}$$

(14d) $$\begin{array}{cc} \; & \;\Delta F_{B}-\Delta F_{B}^{d}=0 \end{array}$$

where L is a Lagrangian function.

The problem (14) is quadratic: it can be efficiently solved by functions in MATLAB, CPLEX, or others. Note that in the *predictor* step, only one iteration of SQP is performed.

#### 3.4.2. *Corrector* Step

By solving Problem (12) in the *predictor* step, we obtained a solution for two angles $\alpha_{F},\alpha_{B}$ and the applied force vector $F_{m}$. The two angles can be applied to the robot; however, with one iteration, $F_{m}$ does not easily satisfy the hard constraint (12c). With two angles, we have a configuration matrix, A; the following optimization problem is solved to find a new applied force vector $F_{m}$:

(15a) $$\begin{array}{cc} \min_{F_{m}} & \;J=∥W_{F}F_{m}{∥}^{2}+{∥W_{B}\left(AF_{m}-F_{B}^{d}\right)∥}^{2} \end{array}$$

(15b) $$\begin{array}{cc} s.t & \;F_{m}\in F \end{array}$$

where $W_{F},W_{B}$ are weighting vectors.

The problem (15) can be considered a classical control allocation problem and be efficiently solved by optimization-based methods or pseudo-based methods [8]. The dead-zone compensation [20] is applied if necessary.

Algorithm 1 shows the procedure to solve the energy-efficient configuration and control allocation problem for our robot.
**Algorithm 1** Energy-efficient and control allocation algorithm.**Input:** Parametric variable $F_{B}^{d}$ (output from the controller) **Output:** Local optimal angles $\alpha_{F}$, $\alpha_{B}$ and applied force vector $F_{m}$    *Predictor* step:
1:Initial guess: primal–dual variable, $\left(x_{k},\lambda_{k},\mu_{k}\right)$; Hessian matrix $\nabla_{x}^{2}L$, and $\nabla_{x}J$, *G*, $\nabla_{x}G$, $\nabla_{x}H$, *H*: all are evaluated at $\left(x_{k},\lambda_{k},\mu_{k}\right)$.2:Solve QP problem (14) and obtain corresponding solution $\left(\Delta x_{k},\tilde{\lambda_{k}},\tilde{\mu_{k}}\right)$3:Compute multipliers direction $\Delta \lambda_{k}=\tilde{\lambda_{k}}-\lambda_{k}$, $\Delta \mu_{k}=\tilde{\mu_{k}}-\mu_{k}$4:Update $x_{k+1}=x_{k}+\Delta x_{k}$, $\lambda_{k+1}=\lambda_{k}+\Delta \lambda_{k}=\tilde{\lambda_{k}}$, and $\mu_{k+1}=\mu_{k}+\Delta \mu_{k}=\tilde{\mu_{k}}$▹ *This is the initial guess for the next time step.*5:Extract two angles $\alpha_{F},\alpha_{B}$ from vector $x_{k+1}$6:Compute configuration matrix, A, corresponding to two angles $\alpha_{F},\alpha_{B}$.  *Corrector* step:7:Solve problem (15) to obtain applied force vector $F_{m}$

**Remark** **3.** *The difference between online optimization and real-time schemes is the initial strategy. This depends on each specific problem. However, in some systems, the initial values are fixed because of limitations or typical applications. For such systems, an offline optimization procedure may be conducted first to find a suitable initial guess. If not, the solution of the current step can be improved in comparison with the previous step. For our dynamically reconfigurable underwater robot, for instance, because of transportation and karst exploration, from the beginning of a mission, the initial configuration of the robot is chosen as $\alpha_{F}=\alpha_{B}=45^{\circ }$. Then, the initial guess is $\alpha_{F}=\alpha_{B}=45^{\circ }$, $F_{m}=0$.*

## 4. Simulation Results

**Figure 4:**
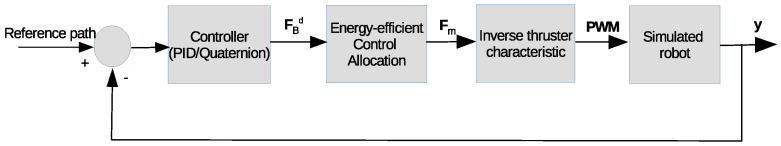
Control loop simulation.

This section presents the simulation results of the proposed approach for our simulated robot. The control diagram of the simulation is shown in Figure 4. A PID or quaternion-based controller was used to derive desired control vector $F_{B}^{d}$. The proposed algorithm inside the energy-efficient control allocation block found the applied force vector, $F_{m}$, corresponding to each desired control vector. The inputs of the thrusters (PWM) were interpolated from vector $F_{m}$ by using inverse thruster characteristics. The next subsection presents more details about the simulated robot and thruster characteristics.

### 4.1. Simulated Robot

**Figure 5:**
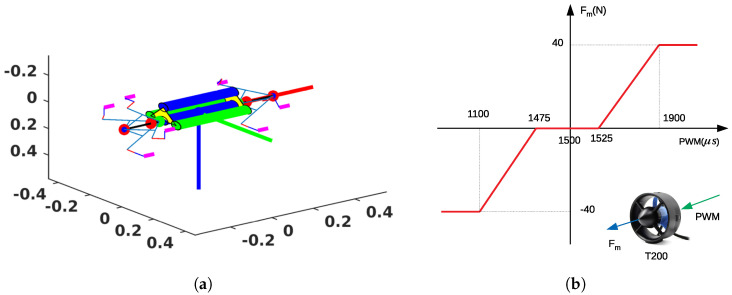
Simulated robot and thruster characteristics. (**a**) Simulated robot, unit of three axis is meter. (**b**) Thruster characteristics.

The simulated robot we built is shown in Figure 5a, which is the same as our prototype robot. Note that in the simulations, external disturbances and model uncertainties were not considered. The robot has three forward thrusters and four backward thrusters. The two blue cylinders are waterproof tubes containing electronic boards, and the two green cylinders are battery tubes. The thruster characteristics are shown in Figure 5b, which approximates the T200 thruster of BlueRobotics [21]. The saturation values of the thrusters are 1100 μs and 1900μs. The dead zone of the thrusters is [1475μs−1525μs]. The robot can vary its configuration. The dynamic model of the robot is simplified as Equation (16). Assume that all feedback states are completely estimated:

(16a) $$\begin{array}{cc} F_{u} & =m_{u}\dot{u}-d_{u}u \end{array}$$

(16b) $$\begin{array}{cc} F_{v} & =m_{v}\dot{v}-d_{v}v \end{array}$$

(16c) $$\begin{array}{cc} F_{w} & =m_{w}\dot{w}-d_{w}w \end{array}$$

(16d) $$\begin{array}{cc} \Gamma_{p} & =m_{p}\dot{p}-d_{p}p \end{array}$$

(16e) $$\begin{array}{cc} \Gamma_{q} & =m_{q}\dot{q}-d_{q}q \end{array}$$

(16f) $$\begin{array}{cc} \Gamma_{r} & =m_{r}\dot{r}-d_{r}r \end{array}$$

where $m_{u},m_{v},m_{w},m_{p},m_{q},m_{r}$ are the total masses (dry mass+added mass or inertia) along each motion axis; $d_{u},d_{v},d_{w},d_{p},d_{q},d_{r}$ are the quadratic damping terms for each motion axis. Note that all coupling terms are neglected. Because the weight of our prototype robot in water is approximately 15 kg, the dynamic parameters of the robot are chosen as $m_{u}=m_{v}=m_{w}=15\;\mathrm{kg},m_{p}=m_{q}=m_{r}=1\;\mathrm{kg},d_{u}=d_{v}=d_{w}=d_{p}=d_{q}=d_{r}=1\;\mathrm{kg}$.

In general, the robot operates with a control loop, in which a controller derives a desired control vector $F_{B}^{d}$. In this section, we describe our simulations of the problem (3) when $F_{B}^{d}$ is the dynamic parameter. We simulated two missions—path-following and station-keeping (observation) problems—which are important in underwater robotics.

### 4.2. Path-Following Problem

For the path-following problem, a line of sight (LoS)-based guidance method [22] was used in this simulation. We compared the energy-like criterion between the two static configurations and the dynamic one in this mission. A PID controller was used in this simulation. The chosen path is a spatial ellipse, which is parameterized as follows:

(17) $$\begin{array}{cc} x & =60\cos(0.2618t) \end{array}$$

(18) $$\begin{array}{cc} y & =60\sin(0.2618t) \end{array}$$

(19) $$\begin{array}{cc} z & =\sin(0.2618t)+5 \end{array}$$

where *t* is a path parameter.

The desired composite speed is $U_{d}=2m/s$. The initial posture of the robot is ${\left[x\left(0\right)\;y\left(0\right)\;z\left(0\right)\;\phi \left(0\right)\;\theta \left(0\right)\;\psi \left(0\right)\right]}^{T}={\left[64\left(m\right)\;3\left(m\right)\;0\;0\;0\;3\pi /4\right]}^{T}$. The initial speed of the AUV is ${\left[u\left(0\right)\;v\left(0\right)\;w\left(0\right)\;p\left(0\right)\;q\left(0\right)\;r\left(0\right)\right]}^{T}={\left[1.5\left(m/s\right)\;0\;0\;0\;0\;0\right]}^{T}$.

To evaluate the efficiency of our approach (dynamic configuration) in comparison with that of others (static configurations), we simulated the path-following problem for the three cases described in Table 1.

**Table 1:** Simulation cases for path-following problem.

No. Case	Two Angles $\alpha_{F},\alpha_{B}$	Notes
1	$\alpha_{F}=\alpha_{B}=70^{\circ }$	Simulation results in Figure 6
2	$\alpha_{F}=\alpha_{B}=90^{\circ }$	Simulation results in Figure 7
3	dynamic	Simulation results in Figure 8

**Figure 6:**
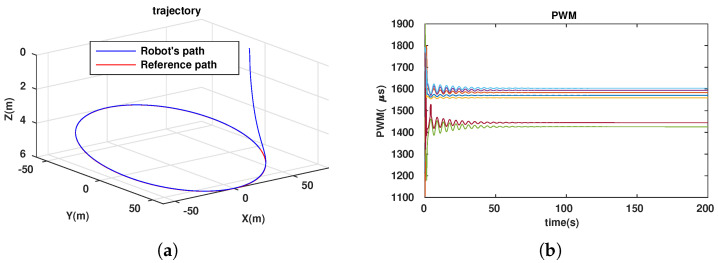
Path following for ellipse with configuration ($\alpha_{F}$ = $\alpha_{B}=70^{0}$). (**a**) Trajectory of robot. (**b**) PWM of 7 thrusters.

**Figure 7:**
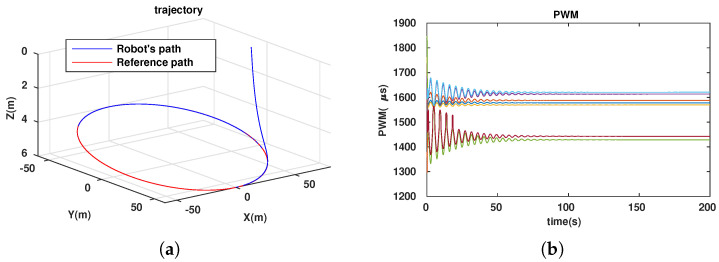
Path following for ellipse with configuration ($\alpha_{F}$ = $\alpha_{B}=90^{0}$). (**a**) Trajectory of robot. (**b**) PWM of 7 thrusters.

**Figure 8:**
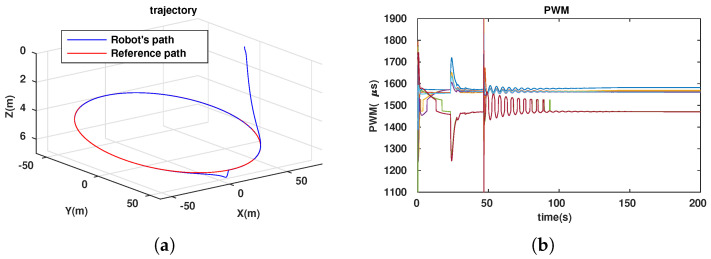

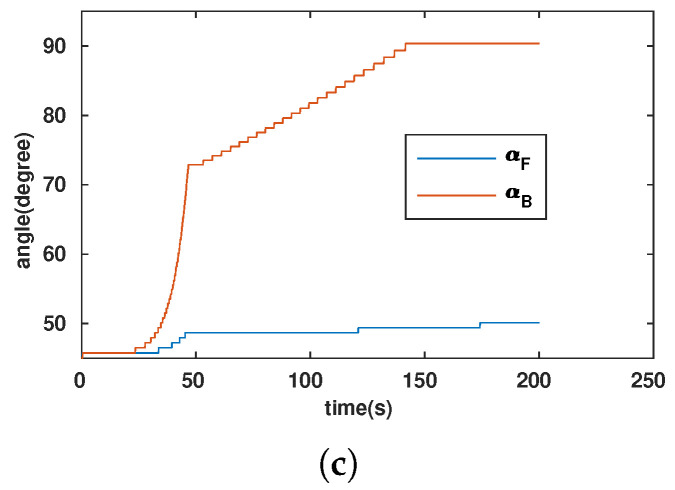
Path following for ellipse with dynamic configuration. (**a**) Trajectory of robot. (**b**) PWM of 7 thrusters. (**c**) Evolution of 2 angles.

**Figure 9:**
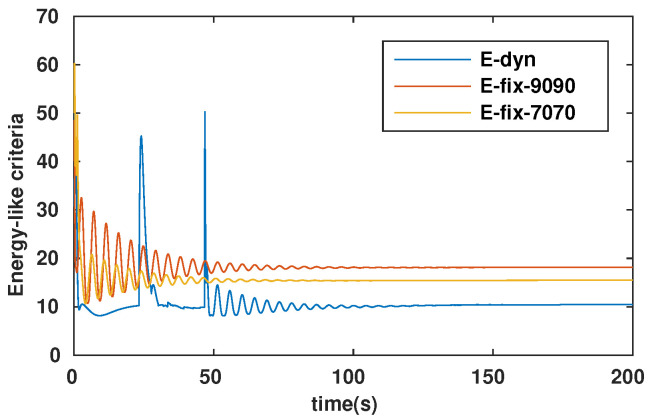
Energy-like criteria for path-following problem.

The energy-like criterion evolutions of the path-following problem for the three simulation cases are shown in Figure 9. We found that the path-following performance was guaranteed for all three cases (see Figure 6a, Figure 7a and Figure 8a). However, with the dynamic configuration, from the energy perspective, the dynamic configuration showed better performance than the other two (see Figure 9). Note that this is only a local optimal solution, and in the configuration space, another optimal solution could exist. For each specific mission, from the initial values ($\alpha_{F}=\alpha_{B}=45^{\circ }$), the robot configuration converges to a local optimal solution. If the mission is suddenly changed, this means that the desired control vector is largely disturbed. This will be carefully investigated and could be an interesting future work, so it is not mentioned in this paper. Following Figure 9, we have two instances at which the energy consumption of the dynamic configuration is larger than the fixed configuration. This happened because some disturbances were injected into the controller to investigate the response of the system with respect to the uncertainties, although this is out of the scope of this paper and will be in the future research. In particular, for the first instance, from 23.4(s) to 27.6(s), a small disturbance was injected into the controller, and for the second one, from 46.8(s) to 46.9(s), a very large disturbance was injected into the controller in a short time. With a small disturbance, the performance of the path-following problem was guaranteed. However, with a very large disturbance, following a path was not guaranteed (a peak in Figure 8a). Indeed, Theorem 1 was violated in this case.

### 4.3. Station-Keeping (Observation) Problem

**Figure 10:**
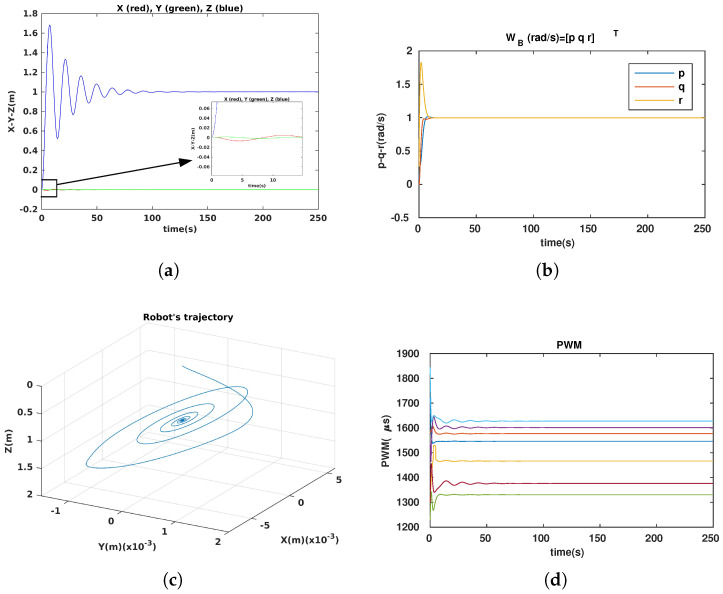
Simulation results with fixed configuration. (**a**) Positions of robot. (**b**) Angular velocities. (**c**) Robot trajectory. (**d**) PWM evolution of 7 thrusters.

**Figure 11:**
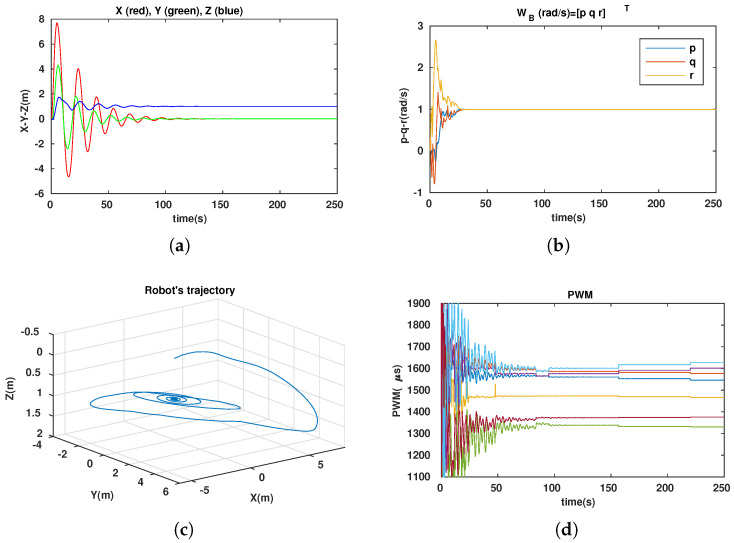

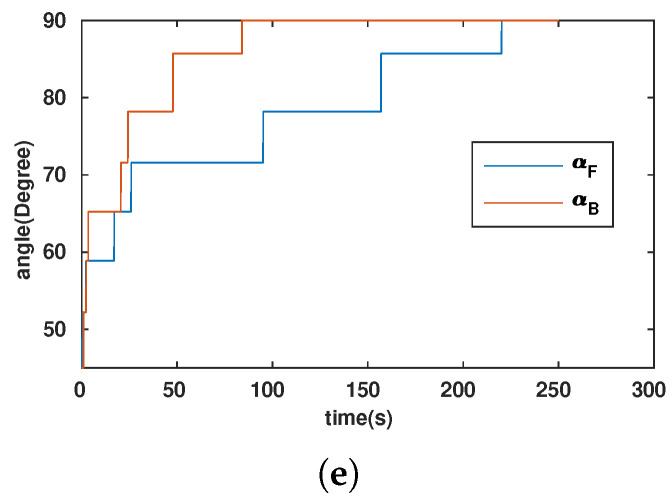
Simulation results with dynamic configuration. (**a**) Positions of robot. (**b**) Angular velocities. (**c**) Robot trajectory. (**d**) PWM evolution of 7 thrusters. (**e**) Two angles’ evolution: $\alpha_{F}$-blue; $\alpha_{B}$-red.

For the station-keeping (observation) problem, the robot normally has to rotate about some DOFs and maintain a constant position, e.g., constant depth. This probably could not be achieved by an under-actuated system, which has some uncontrollable DOFs. Owing to our robot’s versatility, the dynamically reconfigurable robot can easily perform this mission. In this part, we present the simulation results of the observation problem with our robot, in which the robot dove to the desired depth and then rotated with the desired angular velocities, i.e., $\left[x^{d}\;y^{d}\;z^{d}\right]{\left(m\right)}^{T}={\left[0\;0\;1\right]}^{T}$ and $\left[p^{d}\;q^{d}\;r^{d}\right]{\left(rad/s\right)}^{T}={\left[1\;1\;1\right]}^{T}$. The controller was designed with quaternion techniques to avoid singularities (gimbal lock) [23]. The simulations included fixed and dynamic configurations. The simulation results of the fixed configurations, in which $\alpha_{F}=\alpha_{B}=90^{\circ }$, are shown in Figure 10. The simulation results of the dynamic configurations are depicted in Figure 11. Note that in the simulation, we assumed that all states of the robot could be completely measured or estimated.

The control performance was guaranteed in both the fixed and dynamic configurations. The robot reached the desired depth (Figure 10a and Figure 11a) and followed the desired angular velocities (Figure 10b and Figure 11b). As shown in Figure 10d and Figure 11d, the energy-like criterion of fixed configuration was better than that of dynamic configuration because the two angles $\alpha_{F}=\alpha_{B}=90^{\circ }$ could be considered a local optimal solution in this mission (mainly the rotation task). The two angles’ evolution is shown in Figure 11e, which converge to $90^{\circ }$, the same as in the fixed configuration. With initial value $\alpha_{F}=\alpha_{B}=45^{\circ }$, the robot’s configuration continues to improve through the samples. Following Figure 11 (observation problem), the normally rotating priority task is considered. A locally optimal fixed configuration (rotating priority) is chosen. The dynamic configuration converges to the chosen optimal fixed configuration through the time. This shows that our approach drove the system to the locally optimal configuration.

**Remark** **4.** *The proposed method can be used to find the local energy-efficient configuration of a dynamically reconfigurable robot, as described in the previous sections, for problems in which the change in the parametric variable is small enough. However, missions requiring a large change in the parametric variable (the desired control vector) or one DOF to vary from uncontrollable to controllable or vice versa during configuration changes are outside the scope of the proposed method and will be a future research area in terms of system stability and controller design considering a switching mechanism between controllable and uncontrollable DOFs. The experiment we next describe highlights this remark.*

## 5. Experiment Results

**Figure 12:**
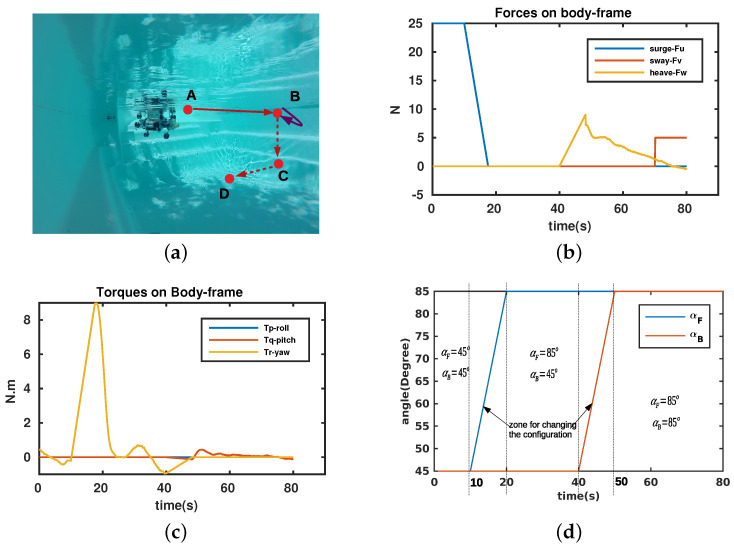
Experiment results. (**a**) Experiment descriptions. (**b**) Desired forces expressed in body frame. (**c**) Desired torques expressed in body frame. (**d**) Evolution of two angles.

**Figure 13:**
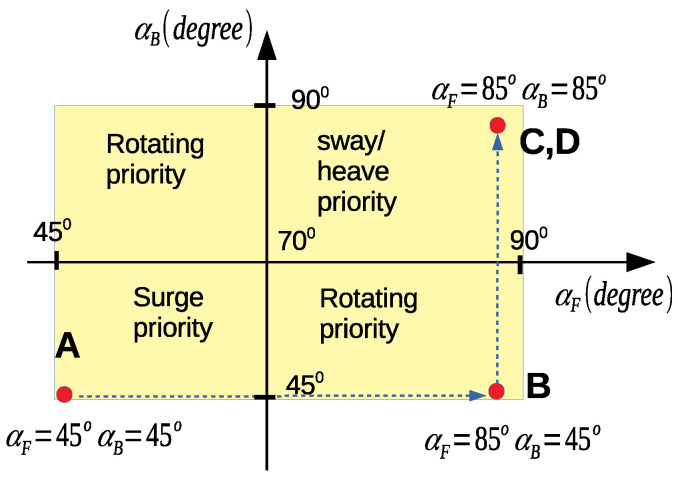
Two angles in feasible map and priority regions.

The real robot was tested in a swimming pool with different configurations. In this test, the robot performed four tasks with two corresponding angles: travel straight (from point *A* to point *B*, $\alpha_{F}=\alpha_{B}=45^{\circ }$), complete a turn $180^{\circ }$ (around point *B*, $\alpha_{F}=85^{\circ },\alpha_{B}=45^{\circ }$), dive to a predefined depth (from point *B* to point *C*, $\alpha_{F}=85^{\circ },\alpha_{B}=85^{\circ }$), and perform a sway (from point *C* to point *D*, $\alpha_{F}=85^{\circ },\alpha_{B}=85^{\circ }$). The robot trajectory is illustrated in Figure 12a. The values of the two angles ($\alpha_{F},\alpha_{B}$) for each trajectory are depicted in Figure 12d. The desired control vector (output from the PID controller), including the desired force and torque elements, is shown in Figure 12b,c. Thanks to our robot, which can vary its configuration by changing two angles $\alpha_{F},\alpha_{B}$, we can divide the feasible space of these two angles into four regions (Figure 13), in which the robot has priority in its DOFs: *surge priority, sway/heave priority*, and *rotating priority*. The term *priority* means that the robot prefers to use such a task in the priority region. For our experiment, the two angles converged to the corresponding priority region.

When we applied the proposed method to this trajectory, with initial values $\alpha_{F}=\alpha_{B}=45^{\circ }$, the trajectory was reasonable and optimal from point *A* to point *B* and when turning around point *B*. Nevertheless, to directly dive from point *B* to point *C*, the robot had to considerably change its configuration enough because the heave DOF is uncontrollable with $\alpha_{F}=\alpha_{B}=45^{\circ }$ and the proximity of these angle values. As such, the robot could not directly perform this dive. So, the robot can use a *hybrid mechanism* between dynamic and static states during its operations. For some parts of the trajectory, the robot can use the dynamic mechanism and, for others, the robot can use a static one with time spent to change the configuration.

## 6. Conclusions and Future Studies

In this paper, we proposed an approach for an energy-efficient configuration and the control allocation of a dynamically reconfigurable underwater robot that was built for karst or confined environment exploration. The energy-like criterion was minimized with respect to the robot constraints. The proposed method was solved online (for each sampling time), corresponding to the robot’s dynamic configuration. The approach was divided into two steps: a *predictor* step and a *corrector* step. In the *predictor* step, the solution of one iteration SQP was chosen for the robot configuration. In the *corrector* step, quadratic programming, as a classical CA method, was solved to adjust the applied force vector that could be assigned for the actuators. The simulation results showed the efficiency of the proposed method through the application of two problems: path following and station keeping (observation). In the future, we will perform real tests with this method. Moreover, external disturbances and model uncertainties will be considered in our subsequent studies.

## Abbreviations

The following abbreviations are used in this manuscript:

AUV	Autonomous Underwater Robot
SQP	Sequential Quadratic Programming
PWM	Pulse Width Modulation
DOF	Degree of Freedom
MPC	Model Predictive Control
CA	Control Allocation
PID	Proportional–Integral–Derivative controller

## Appendix A. Configuration Matrix

The elements of the configuration matrix A are presented in Table A1. Note that *d* and $d_{i},i\in \left\{F,e,t\right\}$ are geometrical distances corresponding to the shape of the robot. $\beta_{i},i\in \left\{1,2,...,7\right\}$ is an angle.

**Table A1:** Elements of **a** matrix.

$u_{1}^{B}=\left(\begin{array}{c} \frac{1}{\sqrt{2}}\left(sin\alpha_{F}+cos\alpha_{F}\right)\\ \frac{\sqrt{3}}{2\sqrt{2}}\left(-cos\alpha_{F}+sin\alpha_{F}\right)\\ \frac{1}{2\sqrt{2}}\left(-cos\alpha_{F}+sin\alpha_{F}\right) \end{array}\right)$	$r_{1}^{B}=\left(\begin{array}{c} \left(d_{F}+L_{F}\right)-\sqrt{d_{e}^{2}+d^{2}}.cos\left(\alpha_{F}+\beta_{1}\right)\\ -\frac{d_{t}}{2}-\frac{\sqrt{3(d_{e}^{2}+d^{2})}sin\left(\alpha_{F}+\beta_{1}\right)}{2}\\ \frac{\sqrt{3}}{2}d_{t}-\frac{\sqrt{d_{e}^{2}+d^{2}}sin\left(\alpha_{F}+\beta_{1}\right)}{2} \end{array}\right)$
$u_{2}^{B}=\left(\begin{array}{c} \frac{cos\alpha_{F}+sin\alpha_{F}}{\sqrt{2}}\\ 0\\ \frac{cos\alpha_{F}-sin\alpha_{F}}{\sqrt{2}} \end{array}\right)$	$r_{2}^{B}=\left(\begin{array}{c} \left(d_{F}+L_{F}\right)-\sqrt{d^{2}+d_{e}^{2}}cos\left(\alpha_{F}+\beta_{2}\right))\\ d_{t}\\ \sqrt{d^{2}+d_{e}^{2}}sin\left(\alpha_{F}+\beta_{2}\right) \end{array}\right)$
$u_{3}^{B}=\left(\begin{array}{c} \frac{1}{\sqrt{2}}\left(sin\alpha_{F}+cos\alpha_{F}\right)\\ \frac{\sqrt{3}}{2\sqrt{2}}\left(cos\alpha_{F}-sin\alpha_{F}\right)\\ \frac{1}{2\sqrt{2}}\left(sin\alpha_{F}-cos\alpha_{F}\right) \end{array}\right)$	$r_{3}^{B}=\left(\begin{array}{c} \left(d_{F}+L_{F}\right)-\sqrt{d_{e}^{2}+d^{2}}.cos\left(\alpha_{F}+\beta_{3}\right)\\ \frac{d_{t}}{2}+\frac{\sqrt{3(d_{e}^{2}+d^{2})}}{2}.sin\left(\alpha_{F}+\beta_{3}\right)\\ \frac{\sqrt{3}}{2}d_{t}-\frac{\sqrt{d_{e}^{2}+d^{2}}}{2}sin\left(\alpha_{F}+\beta_{3}\right) \end{array}\right)$
$u_{4}^{B}=\left(\begin{array}{c} \frac{cos\alpha_{B}+sin\alpha_{B}}{\sqrt{2}}\\ \frac{-cos\alpha_{B}+sin\alpha_{B}}{\sqrt{2}}\\ 0 \end{array}\right)$	$r_{4}^{B}=\left(\begin{array}{c} -(d_{F}+\sqrt{d^{2}+d_{e}^{2}}cos\left(\alpha_{B}+\beta_{4}\right))\\ -(\sqrt{d^{2}+d_{e}^{2}}sin\left(\alpha_{B}+\beta_{4}\right))\\ -dt \end{array}\right)$
$u_{5}^{B}=\left(\begin{array}{c} \frac{cos\alpha_{B}+sin\alpha_{B}}{\sqrt{2}}\\ 0\\ \frac{cos\alpha_{B}-sin\alpha_{B}}{\sqrt{2}} \end{array}\right)$	$r_{5}^{B}=\left(\begin{array}{c} -(d_{F}+\sqrt{d^{2}+d_{e}^{2}}cos\left(\alpha_{B}+\beta_{5}\right))\\ d_{t}\\ \sqrt{d^{2}+d_{e}^{2}}sin\left(\alpha_{B}+\beta_{5}\right) \end{array}\right)$
$u_{7}^{B}=\left(\begin{array}{c} \frac{cos\alpha_{B}+sin\alpha_{B}}{\sqrt{2}}\\ 0\\ \frac{-cos\alpha_{B}+sin\alpha_{B}}{\sqrt{2}} \end{array}\right)$	$r_{7}^{B}=\left(\begin{array}{c} -(d_{F}+\sqrt{d^{2}+d_{e}^{2}}cos\left(\alpha_{B}+\beta_{7}\right))\\ -d_{t}\\ -(\sqrt{d^{2}+d_{e}^{2}}sin\left(\alpha_{B}+\beta_{7}\right)) \end{array}\right)$

## Appendix B. Notations

This section describes the notations used in the paper. However, further notations are introduced when needed.

A	Configuration matrix
$u_{i}^{B}$	(3×1) unit vector of direction of the ith thruster with respect to body frame
$r_{i}^{B}$	(3×1) unit vector of position of the ith thruster with respect to body frame
$F_{m}$	(m×1) applied force vector of *m* thrusters
$F_{m,i}$	Applied force magnitude of the ith thruster
$F_{B}^{d}$	(6×1) desired control vector (including force and torque) with respect to
	body frame
FB=fτ	(6×1) resulting control vector (including force and torque) with respect to
	body frame
⊗	Cross product
∥·∥	Euclidean norm
*m*	Number of thrusters
*n*	Number of degrees of freedom (DOFs)
